# Mannitol ingestion causes concentration-dependent, sex-biased mortality in adults of the fruit fly (*Drosophila melanogaster*)

**DOI:** 10.1371/journal.pone.0213760

**Published:** 2019-05-31

**Authors:** Katherine Fiocca, Meghan Barrett, Edward A. Waddell, Jennifer Viveiros, Cheyenne McNair, Sean O’Donnell, Daniel R. Marenda

**Affiliations:** 1 Department of Biology Drexel University, Philadelphia, PA, United States of America; 2 Department of Biodiversity, Earth and Environmental Science, Drexel University, Philadelphia, PA, United States of America; 3 Department of Neurobiology and Anatomy, Drexel University College of Medicine, Philadelphia, PA; Durham University, UNITED KINGDOM

## Abstract

Mannitol, a sugar alcohol used in commercial food products, has been previously shown to induce sex-biased mortality in female *Drosophila melanogaster* when ingested at a single concentration (1 M). We hypothesized that sex differences in energy needs, related to reproductive costs, contributed to the increased mortality we observed in females compared to males. To test this, we compared the longevity of actively mating and non-mating flies fed increasing concentrations of mannitol. We also asked whether mannitol-induced mortality was concentration-dependent for both males and females, and if mannitol’s sex-biased effects were consistent across concentrations. Females and males both showed concentration-dependent increases in mortality, but female mortality was consistently higher at concentrations of 0.75 M and above. Additionally, fly longevity decreased further for both sexes when housed in mixed sex vials as compared to single sex vials. This suggests that the increased energetic demands of mating and reproduction for both sexes increased the ingestion of mannitol. Finally, larvae raised on mannitol produced expected adult sex ratios, suggesting that sex-biased mortality due to the ingestion of mannitol occurs only in adults. We conclude that sex and reproductive status differences in mannitol ingestion drive sex-biased differences in adult fly mortality.

## Introduction

D-mannitol (henceforth mannitol) is a 6-carbon polyol produced via microbial fermentation, particularly by yeasts, and is the most common naturally-occurring polyol in plants and fungi [1–4]. Mannitol is commonly used as a sweet additive in consumer products as it is only partially absorbed in the human small intestine without increasing insulin secretion or blood glucose [1,5].

Mannitol produces a variety of gastrointestinal, reproductive, and survival effects when fed to other organisms [2,6,7]. For example, mannitol reduced survival and prevented adult female reproduction in *Pimpla turionellae* ichneumonid wasps [7]. In contrast to *P*. *turionellae*, mannitol stimulated feeding behavior at low doses (72.6 mM) and increased the longevity of females in comparison to males in red flour beetles (*Tribolium castaneum*) [8,9]. Our previous work has shown that adult female *Drosophila melanogaster* fed media containing 1 M mannitol showed significantly decreased longevity over a seventeen-day trial in comparison to males [10].

We hypothesized that the sex-biased effects of mannitol in *D*. *melanogaster* could be caused by differing energetic demands between males and females. Oogenesis requires greater protein intake [11,12], leading females to eat more food, more frequently [13,14]. Sex-biased differences in survival between males and females could be due to differences in ingestion that increase self-dosing of mannitol in females. To test this hypothesis, first we assessed if mortality was concentration dependent in both males and females, suggesting dose-dependency, and if sex-biased differences in survival were consistent across concentrations. We also assessed if food consumption was higher for female flies fed mannitol, versus male flies fed mannitol, using the CAFE assay.

Next, we determined if flies differed in survival when cultured in single sex vials or when cultured in vials with the opposite sex (mixed-sex). Prior studies showed female fly feeding rates increase substantially when cultured, and mated, with males [15–17]. Additionally, reproduction can also be energetically costly for males due to courtship, competition, and mating behaviors, as well as sperm production [18–22]. Therefore, we predicted that both female and male mortality would be higher in mixed-sex vials as compared to single-sex vials. To separate the effects of being mated from being cultured continuously with the opposite sex, we compared the longevity of virgins housed in single-sex vials, mated flies housed in single-sex vials, and continuously mating flies in mixed-sex vials.

To test whether increased female mortality was inherently related to sex differences, such as genetic effects, we tested whether this sex-bias also occurred during the immature developmental stages. We reared larvae on media containing increasing concentrations of mannitol and found no significant difference in the eclosion of each sex (adult sex ratio). Our results suggest that sex-biased mortality is consistent across concentrations in adults only. Male lethality is amplified only when male and female flies are cultured together, while female lethality is amplified after mating, regardless of culturing condition. Finally, we explore possible mechanisms of mannitol-induced mortality.

## Materials and methods

### Culturing *Drosophila*

Wild-type (Canton S, Bloomington Drosophila Stock Center) *D*. *melanogaster* were raised to adulthood on standard *Drosophila* media for laboratory culturing and reared in an insect growth chamber at 25˚C, 50% relative humidity, with a 12-h:12-h photoperiod [23]. Media were prepared in 100 ml batches as follows: 9.4 g cornmeal, 3.77 g yeast, 0.71 g agar, 0.75 ml Propionic acid, 1.88 ml Tegosept (10% w/v methyl p-hydroxybenzoate in 95% ethanol), 0.05% Brilliant Blue R-250 dye, and distilled water to a final volume of 100 ml. Control media (containing no mannitol) consisted of the treatment recipe with and without 9.42 ml of molasses (Genesee Scientific). Mannitol treatment media consisted of the treatment ingredients as well as treatment-specific amounts of mannitol (HiMedia; GRM024-500G, Lot 000249743). After heating the mixed ingredients to set the agar, media were poured into vials and cooled until consistency was firm and uniform. An excess of media was provided, with 10 ml per vial.

### Testing concentration-dependent sex-biased adult mortality in single and mixed-sex vials

Adult flies that were 0–24 hours post-eclosion were transferred from the standard media to the treatment media. Control media, with and without (0 M) molasses, and treatment media with mannitol concentrations of 0.25 M, 0.5 M, 0.75 M, 1 M and 2 M were used. Three vials of 10 female flies, three vials of 10 male flies, and three vials of 5 female and 5 male flies were used per treatment (n = 90 flies/treatment). Flies were moved to fresh treatment vials of the same formulation every four days. Every 24 hours for 21 days, dead flies were removed from their vials, counted, and sexed. Dead flies were also dissected under a dissecting scope, to look for the presence of blue dye or other artefacts of mannitol ingestion in the crop. Photos of representative adult flies were taken on day four of mannitol ingestion from additional 0 M (no mannitol) and 0.75 M treatment vials set up specifically for photos, after the initial round of dissections and data collection.

### Testing adult mortality based on culturing condition and mating status

Virgin adult flies that were 0–24 hours post-eclosion were kept on standard media in single-sex vials for three days. On day 3, pairs of female and male fly were mated together and, following confirmation of mating, moved to single-sex vials containing either 0 M or 0.75 M mannitol treatment media (n = 10 flies/vial, three vials/treatment). Six vials of 5 female and 5 male flies, three vials of 10 virgin females, and three vials of 10 virgin males were also used per treatment. Flies were moved to fresh treatment vials of the same formulation every four days. Every 24 hours for 21 days, dead flies were removed from their vials, counted, and sexed. Dead flies were also dissected under a dissecting scope, to look for the presence of blue dye or other artefacts of mannitol ingestion in the crop. Photos of representative adult flies were taken on day four of mannitol ingestion from additional 0 M (no mannitol) and 0.75 M treatment vials set up specifically for photos, after the initial round of dissections and data collection.

### Testing consumption in male and female flies fed mannitol versus sucrose

CAFE experiments were performed to assess differences in food consumption between male and female flies fed mannitol and between flies of each sex fed sucrose versus mannitol, using previously established methods [24,25]. Trials were performed at 23 ˚C and 40% relative humidity. Flies were held in shortened culturing vials that are 7 cm in length. 5 ml of 1% agar was poured into the bottom of each vial for humidity and cotton flugs were inserted 1 cm deep, which was then parafilmed to reduce evaporation and keep humidity high inside the vials. Microcapillary tubes were first dipped in mineral oil before being filled with a solution of either 5% mannitol or 5% sucrose w/v, and both 5% yeast extract w/v and 0.1% Brilliant Blue FCF dye (to confirm consumption). Microcapillary tubes were inserted into the tube through a 200 μl pipette tip in the cotton flug. One microcapillary tube, either mannitol or sucrose condition, was used per vial.

0–24 hour old virgin flies were collected and held separately for three days. On day 3, pairs of male and female flies were mated together and, after confirmation of mating, separated back into single-sex vials. On day four, flies were separated into CAFE vials, with five flies of a sex in a vial (15 flies/sex/condition; n = 120 flies). Three vials per condition were prepared with no flies, as an evaporation control. Fly consumption was documented after five hours by using a digital caliper to measure the amount of liquid consumed from the tube (in mm). To control for evaporation, the three evaporation vials for that condition were averaged and the average was subtracted from each experimental tube. Final measurements were divided by 25 (5 flies over 5 hours) to get the consumption per fly per hour. Any experimental vials where less food was consumed than the smallest evaporation control vial were considered to have failed and were not used in the analysis (25% of sucrose vials, 0% of mannitol vials).

### Testing effects of feeding mannitol to larvae on adult sex ratios and eclosion time

Groups of 15 male and 15 female wild-type flies raised on standard media were placed in vials containing 0M, 0.4M, or 0.8M mannitol treatment media and allowed to mate and lay eggs for 24 hours before the adults were removed. Nine vials were used per concentration, with a total of 405 flies of each sex used for laying. Vials were checked for newly eclosed adults every twelve hours from Day 10 to Day 15, and every twenty-four hours from Day 15 to Day 24 (when relatively few adults eclose). Adult flies were removed from the vials and sexed, and their eclosion day was recorded to the nearest 24 hours (0M: n = 904; 0.4M: n = 1264; 0.8M: n = 262 adults).

### Statistical analyses

Analyses were performed using SPSS v. 24 software, Graphpad Prism v. 8.0.0, and Sigmaplot v. 12.5 [26–28]. Sex-biased adult longevity to 21 days across concentrations was assessed using survival analyses in SPSS [29], with subjects living to the end of the trial or lost to reasons other than death (i.e., escaped or injured in handling) included in the analysis as right-censored values. Differences in survival distributions across concentrations, by culturing condition (single vs. mixed sex), by mating status and culturing condition (virgin and single sex, mated and single-sex, mated and mixed-sex), and by sex (for adults) were tested using pairwise log-rank Mantel Cox survival analysis tests. Mean pr(mortality) and standard error were calculated for each concentration. A three-parameter sigmoid curve was fitted to survival data from all females in Sigmaplot to assess adult female LC_50_ at 21 days; male LC_50_ could not be calculated as males did not reach 50% mortality at 21 days at any tested mannitol concentration.

Differences in the consumption of male vs. female flies fed mannitol and differences in the consumption of mannitol vs. sucrose fed flies of each sex in the CAFE were tested using a One-Way ANOVA in GraphPad with a Sidak’s multiple comparisons test. Differences in adult sex ratios were tested using a chi square in GraphPad against an expected 50–50 male-female ratio [30], with the same sample size as in the treatment vial. All treatment vial sex ratios were also tested against our control vials using a chi square. Differences in mean time to eclosion of males and females across concentrations were assessed using a pairwise log-rank Mantel Cox survival analysis test. Linear regressions of male and female eclosion day vs. mannitol concentration, with comparisons of slopes and intercepts, were used to determine if the concentration of mannitol had a similar effect on eclosion day in both sexes.

## Results

### Concentration-dependent, sex-biased adult longevity to 21 days

Flies fed control media without molasses did not significantly differ in their longevity to 21 days from those fed control media with molasses (X^2^ = 0.2, p = 0.66), demonstrating that this source of carbohydrates was not necessary for adult *D*. *melanogaster* survival to 21 days. Given this finding, all further results utilized the ‘no molasses’ control treatment as the comparison 0 M control.

**Fig 1:**
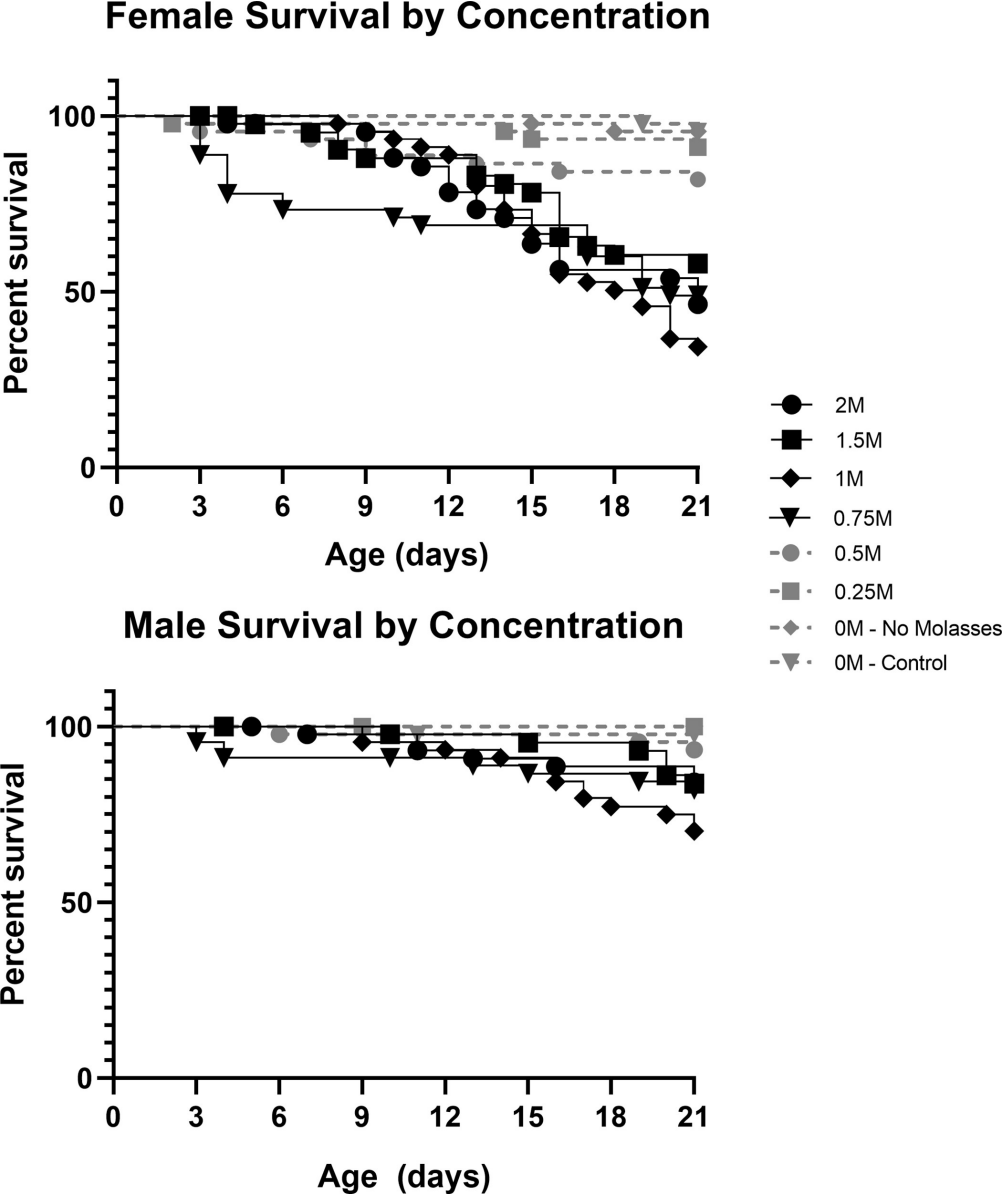
Males and females fed mannitol have concentration-dependent decreases in survival. Survival plots showing percent survival versus adult age of female (top) and male (bottom) *D*. *melanogaster* fed control media or media with increasing concentrations of mannitol (0.25 M to 2 M). Observations were terminated at 21 days of age (n = 45flies/sex/treatment). Highly significant differences (p<0.01) from the control are in black, non-significant differences are in grey.

Longevity over 21 days was dependent on mannitol concentration for both sexes. Female longevity did not significantly differ between the controls and the 0.25 M treatment (X^2^ = 0.73, p = 0.39), but differed significantly in all other mannitol treatments (Fig 1; 0.5 M: X^2^ = 4.30, p = 0.04; 0.75 M: X^2^ = 24.84, p<0.001; 1 M: X^2^ = 37.88, p<0.001; 1.5 M: X^2^ = 17.82, p<0.001; 2 M: X^2^ = 26.788, p<0.001). Male longevity to 21 days did not differ significantly between the controls and the 0.25 M (no difference observed) or 0.5 M (X^2^ = 3.07, p = 0.08) treatments, but differed significantly from the other mannitol treatments (Fig 1; 0.75 M: X^2^ = 8.81, p = 0.003; 1 M: X^2^ = 15.60, p<0.001; 1.5 M: X^2^ = 7.86, p = 0.005; and 2 M: X^2^ = 7.69, p = 0.006).

The best-fit sigmoidal curve for adult female LC_50_ data at 21 days was:

$$\mathrm{Pr}\left(adult\;female\;mortality\right)=0.5439/(1+e^{\left(\frac{-([Mannitol]-0.5474)}{0.0856}\right)})$$

This curve was a significant fit to the data (S1 Fig; R^2^ = 0.898, p = 0.013) and using the equation we estimated the adult female LC_50_ at 21 days to be 0.76 M mannitol. Males did not have an LC_50_ in this experiment; maximum adult male mortality at 21 days was 30.2%, in the 1 M mannitol treatment.

**Fig 2:**
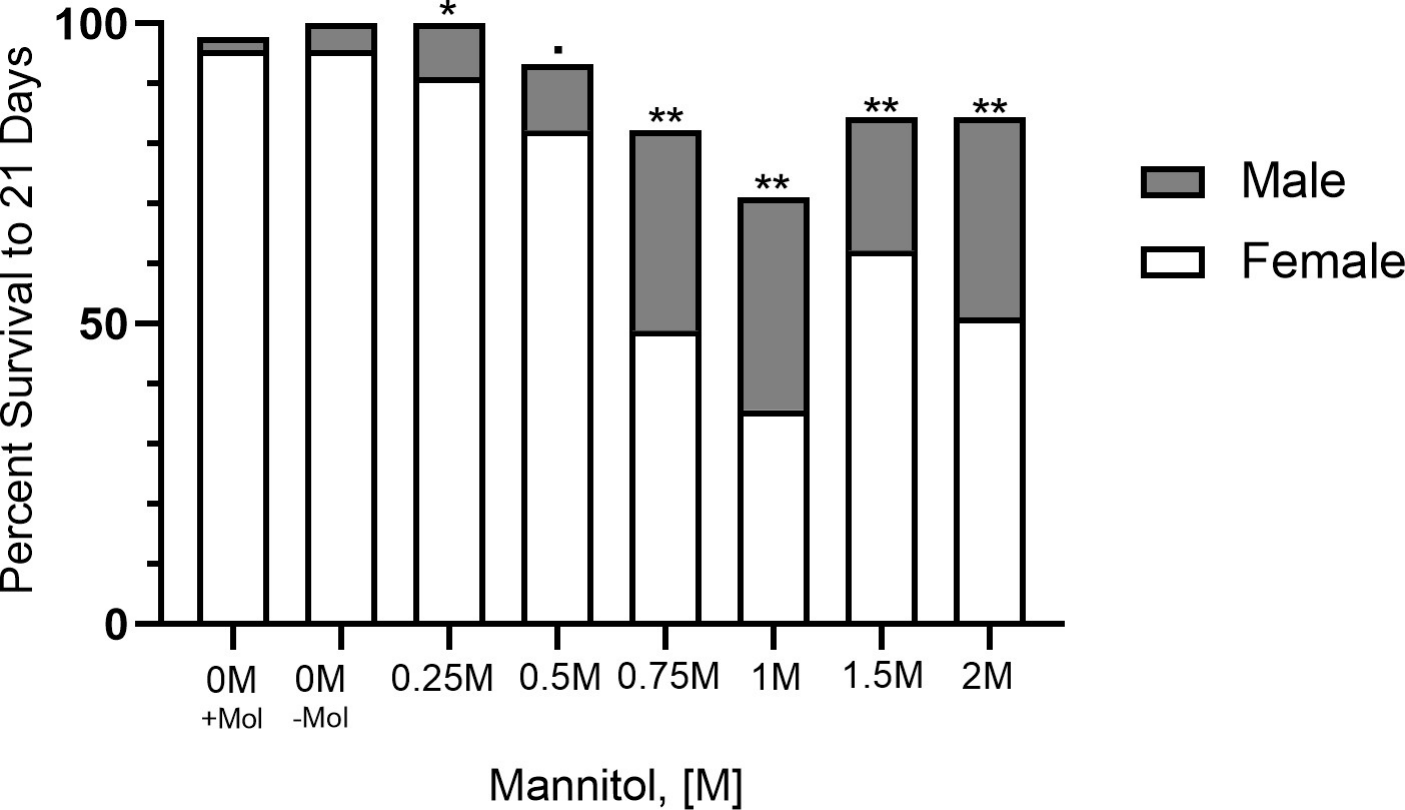
Females experience reduced survival compared to males when fed mannitol at high concentrations. Plot showing the difference in percent survival of male flies versus female flies when fed control media or media with varying concentrations of mannitol (0.25 M to 2 M). Observations were terminated at 21 days of age (n = 45flies/sex/treatment). Highly significant differences in survival distributions (Mantel-Cox; p<0.01) are denoted by two stars, significant differences (p<0.05) are denoted by one star, nearly significant differences (p<0.1) are denoted by a dot, and non-significant differences have no symbols.

Adult male and female flies did not differ in their longevity to 21 days in either 0 M condition (molasses: X^2^ = 0.33, p = 0.57; no molasses: X^2^ = 2.02, p = 0.16) or the 0.5 M mannitol treatment (X^2^ = 2.79, p = 0.09). The sex difference was marginally significant in the 0.25 M treatment (X^2^ = 4.07, p = 0.04). Adult females had highly significantly decreased longevity relative to males in the other treatments (Fig 2; 0.75 M: X^2^ = 10.65, p = 0.001; 1 M: X^2^ = 12.13, p<0.001; 1.5 M: X^2^ = 7.92, p = 0.005; 2 M: X^2^ = 13.54, p<0.001).

### Food consumption of male and female mannitol-fed, vs. sucrose-fed, flies using a CAFE assay

Female flies fed a 5% mannitol diet consumed more media per hour than male flies fed mannitol (S2 Fig; n = 30 flies/sex, Sidak’s MCT; p = 0.0035), confirming differences in food consumption between the sexes reported in the literature [13,14]. No difference was found in female or male consumption between sucrose-fed and mannitol-fed flies (Sidak’s MCT; female: p = 0.15, male: p = 0.80).

### Dye excretion and matter accumulation in the crops of adult flies

**Fig 3:**
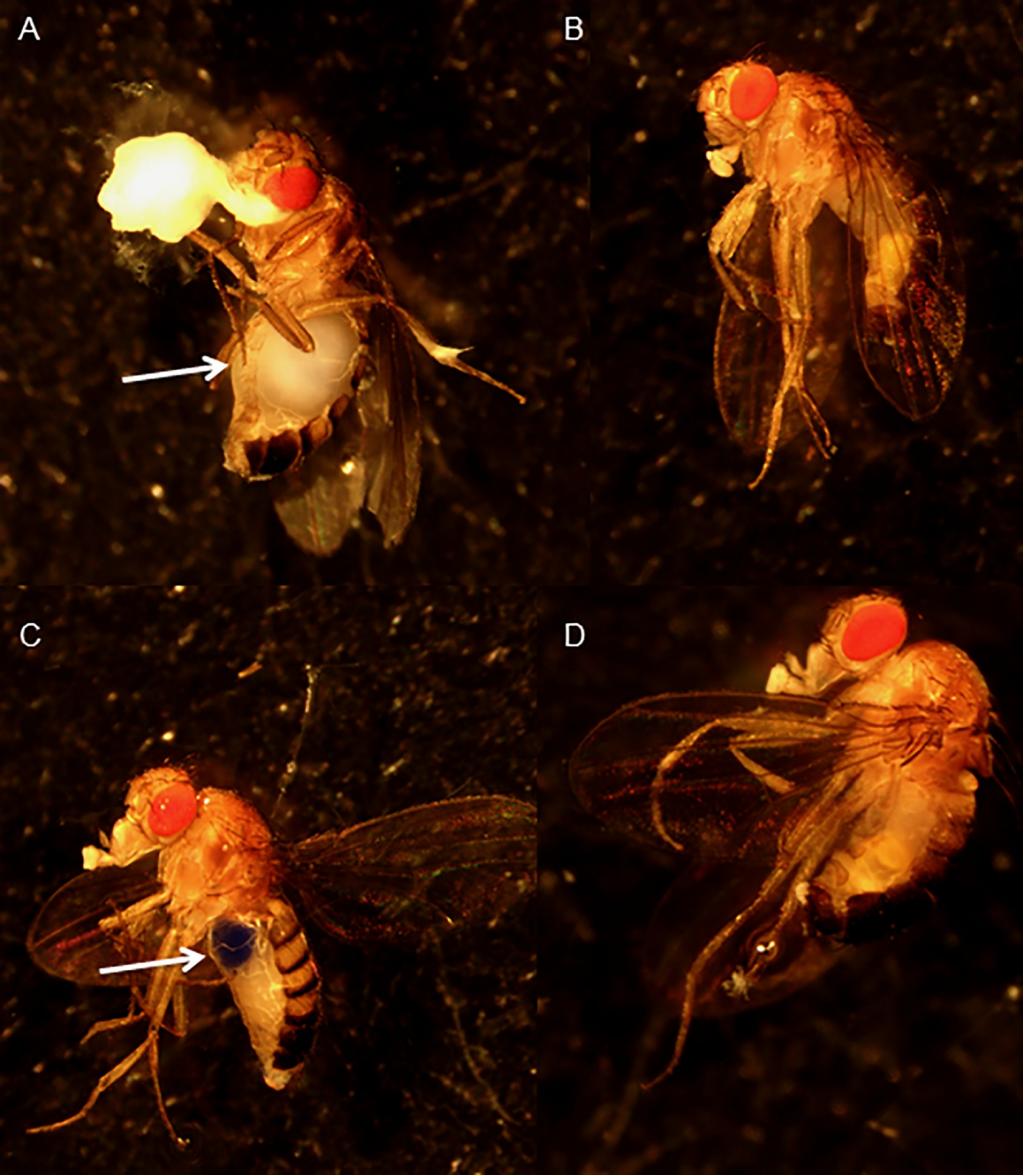
Deceased mannitol-treated females had white masses in their crop. Images of adult Canton S flies after four days of exposure to treatment media. A) Female fed 0.75 M mannitol and found dead in treatment before photographing. Arrow indicates accumulated white mass in the crop. B) Male fly sacrificed for photograph and was found alive in the 0.75 M mannitol treatment vial. C) Female sacrificed from control. Arrow indicates blue dye in the crop. D) Male sacrificed for photograph from control.

Blue dye was found in nearly 100% of flies by visual inspection on day 1 of trials. Additionally, fecal matter was blue in all treatment vials at all time points, indicating that the flies consumed the mannitol-containing media throughout the experiment. However, dissection of deceased mannitol-treated females revealed large amounts of white matter within the crop, and often also around the mouth and anus, as early as day four in the 0.75 M trials; this was not true of living 0 M females sacrificed at the same time point (Fig 3). Deceased mannitol-treated males also have white matter within the crop upon dissection, though the amount was significantly less and did not cause the visible distension of the abdomen seen in females.

No blue dye was found in the crops of dead, mannitol-treated flies suggesting the dye was excreted while other components of the media remained in the crop. Indigestible dyes like Brilliant Blue R-250 will begin to be excreted as early as 15 minutes after ingestion, and are completely gone from the fly’s digestive tract after 24 hours [31,32]. Overall, this observation suggests that some components of the mannitol-treated media, like the dye, are able to pass through the digestive tract while other material remains and accumulates in the crop around the time of death.

### Differences in longevity to 21 days in mixed sex vs. single sex vials

**Fig 4:**
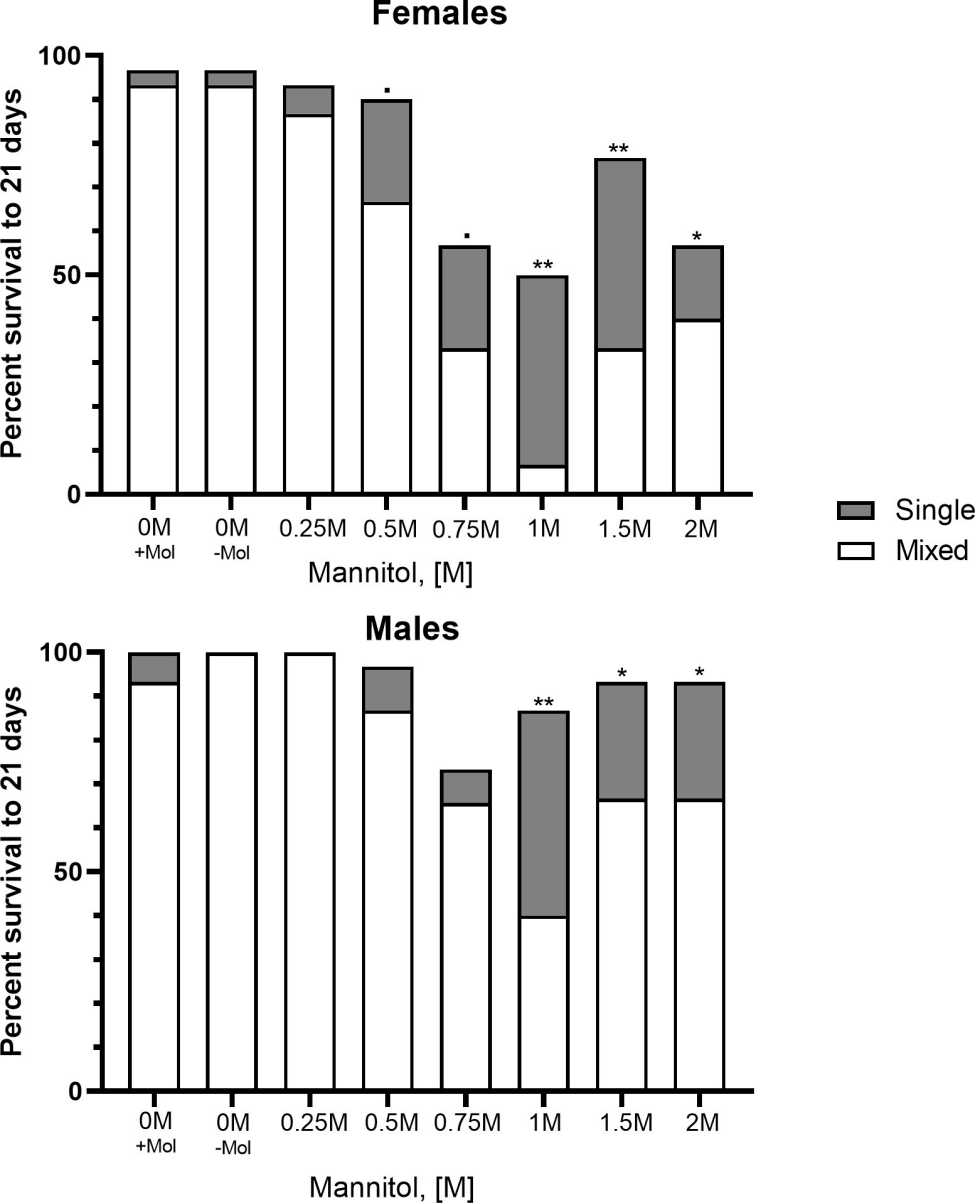
Continuously mating flies have reduced survival compared to non-mating flies of same sex at high mannitol concentrations. Plot showing the difference in percent survival of flies cultured in single sex vials versus flies of the same sex cultured in mixed sex vials when given media with the same concentration of mannitol. Observations were terminated at 21 days (n = 30 flies/sex for single sex treatments; n = 15 flies/sex for mixed-sex treatments). Highly significant differences in survival distributions (Mantel-Cox; p<0.01) are denoted by two stars, significant differences (p<0.05) are denoted by one star, nearly significant differences (p<0.1) are denoted by a dot, and non-significant differences have no symbols.

We next determined whether being cultured with or without access to the opposite sex affected longevity of males or females. Females flies in mixed-sex vials had significantly reduced longevity to 21 days compared to females kept in single-sex vials in the 1 M, 1.5 M, and 2 M treatments (Fig 4; 1 M: X^2^ = 8.67, p = 0.003; 1.5 M: X^2^ = 9.73, p = 0.002; 2 M: X^2^ = 4.12, p = 0.04) but not in the control, nor in the lower concentration treatments (0 M: X^2^ = 0.28, p = 0.6; 0.25M: X^2^ = 0.46, p = 0.5; 0.5 M: X^2^ = 3.33, p = 0.07; 0.75 M: X^2^ = 2.83, p = 0.09). A similar pattern was observed for males (Fig 4; 1 M: X^2^ = 12.65, p<0.001; 1.5 M: X^2^ = 5.80, p = 0.02; 2 M: X^2^ = 5.92, p = 0.02; 0 M: X^2^ = 2.00, p = 0.16; 0.25 M: X^2^ = n.d., p = n.d.; 0.5 M: X^2^ = 1.50, p = 0.22; 0.75 M: X^2^ = 0.93, p = 0.33). In both cases, the majority of the difference in survival between single-sex vials and mixed-sex vials occurred after 12–15 days (S3 Fig).

### Effects of culturing condition and mating status on adult male and female mortality

**Fig 5:**
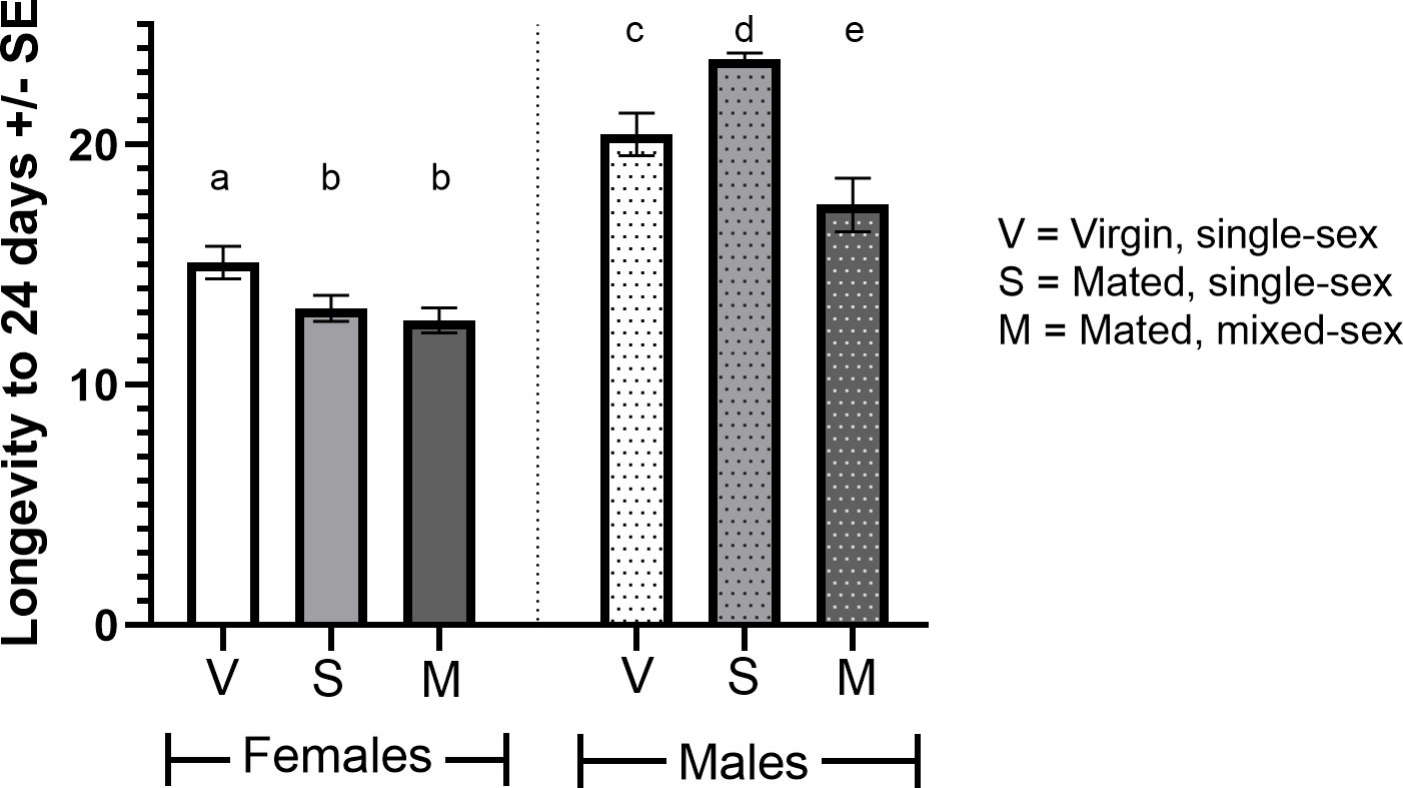
Effects of culturing condition and mating status on adult male and female mannitol-induced decreases in longevity. Plot showing the difference in longevity to 24 days (21 trial days + 3 days on standard media) of virgin adults fed 0.75 M mannitol cultured in single-sex conditions, mated adults cultured in single-sex conditions, and mated adults cultured in mixed-sex conditions (n = 30/sex/treatment). Significant differences in survival distributions (Mantel-Cox; p<0.05) are denoted by different letters.

In the previous experiment, males and females in single-sex vials were likely to be virgins as they were only housed with the opposite sex for up to 24 hours post-eclosion (a period in which both sexes exhibit low receptivity to mating [33–35]). However, we further tested whether mating status (virgin or mated) or culturing condition (single-sex or mixed-sex vials) had an effect on adult mortality. Mated, 0.75 M mannitol-fed females did not differ in their longevity when cultured in single-sex or mixed-sex conditions (Fig 5; X^2^ = 0.26, p = 0.61), however both differed significantly from virgin females kept in single-sex conditions (mated, single-sex: X^2^ = 7.59, p = 0.006; mated, mixed-sex: X^2^ = 11.84, p = 0.001). Mated, 0.75 M mannitol-fed males differed in their longevity when cultured in single-sex or mixed-sex conditions (Fig 5; X^2^ = 23.00, p<0.001), and virgin males differed from mated males in single-sex conditions (X^2^ = 7.99, p = 0.005) and mixed-sex conditions (X^2^ = 4.824, p = 0.028).

### Effects of mannitol fed to larvae on adult sex ratios and eclosion day

We asked whether larvae fed mannitol would experience the same sex-biased mortality found in adults. The expected adult sex ratios upon eclosion are 1:1 [30]. None of our conditions deviated from the expected ratio (Chi square, 0 M: p = 0.71; 0.4 M: p = 0.13; 0.8 M: p = 0.73), nor did treatment conditions deviate from the sex ratio of our 0 M condition (Chi square: p = 0.20). Eclosion day was significantly later in both males and females in 0.4 M trials compared to controls (S4 Fig: Mantel-Cox, males: X^2^ = 308.51, p<0.001; females: X^2^ = 491.21, p<0.001) and even more delayed in 0.8 conditions than 0.4M (Mantel-Cox, males: X^2^ = 401.47, p<0.001; females: X^2^ = 448.98, p<0.001). However, there was no significant difference in developmental delay between females and males (S5 Fig: slopes: p = 0.14; elevations: p<0.0001) and, as expected [36], males in all conditions emerged later than females at their concentration (Mantel-Cox, p<0.001).

## Discussion

This study aimed to determine if mannitol’s effects were consistently sex-biased in adults and larvae across increasing concentrations of mannitol, and if mating status or culturing condition affected mortality. Mortality induced by mannitol was concentration-dependent for both sexes. Female adult flies ingesting mannitol showed significant decreases in longevity to 21 days compared to controls from 0.5 M-2 M mannitol, while males raised on 0.75 M-2 M mannitol showed significant decreases in longevity compared to controls. Females had an LC_50_ of 0.76 M at 21 days, while males reached a maximum mortality of 30.2% as a result of 1 M mannitol treatment. These decreases in longevity to 21 days were concentration-dependent at concentrations below 1 M mannitol for both sexes. Survival was somewhat rescued at the highest concentrations of mannitol tested (1.5 M and 2 M) for both males and females. A potential explanation for this rescue could be that flies detect the presence of mannitol at these extreme concentrations, which may decrease palatability. Alternatively, media was observed to become crystalline at these higher concentrations, which may deter normal consumption and reduce mortality. It is important to note that flies were still able to consume and excrete media at these concentrations given the presence of blue dye in their crops (at 24 hours) and fecal matter (throughout the experiment).

Decreases in adult female longevity to 21 days were greater than that of males when treated with mannitol at concentrations of 0.75 M and above. Our results supported our hypothesis that increased food ingestion, and therefore self-dosing with mannitol, due to differing reproductive costs generates sex-biased differences in mortality between males and females. Males and females have differing nutritional requirements generated by the different energetic demands of reproduction [20]. Females have been reported to feed more frequently, and consume greater volumes of food, than males because oogenesis is both energetically and nutritionally costly [13,14]. In our experiment, females fed mannitol were found to consume more media per hour than male flies fed mannitol, replicating previous results from the literature.

Differences in longevity to 21 days between single-sex and mixed-sex vials may also be due to reproductive energetic demands that generate differences in food ingestion and self-dosing between members of the same sex. Females and males housed in mixed-sex vials had significantly decreased longevity compared to those kept in single-sex vials at concentrations of 1 M, 1.5 M, and 2 M mannitol. This is likely due to the incidences of copulation and reproduction, both of which are energetically costly for either sex [37–39]. Fertile *D*. *melanogaster* females feed more often than sterile or virgin females and the receipt of seminal proteins (sex peptides) also induced feeding and reproduction in fertile females [15,40]. This is consistent with our results that mating, not culturing condition, influences ingestion and thus mortality in females.

*Drosophila bifurca* males raised with infrequent access to females produced significantly less sperm than those in mixed-sex conditions [39]. This is consistent with our results that males kept in single-sex vials (even if mated once) have reduced longevity compared to males in mixed-sex vials. Behaviors associated with courtship, competition, and mating may also be energetically costly for males, causing increased food ingestion, and thus mannitol self-dosing, for males in mixed-sex vials [18–20,22].

Female longevity has been shown to decrease with increased male encounter rates and with exposure to male seminal proteins [30,41], however females in mixed-sex control vials did not differ in their longevity from females in single-sex control vials. Additionally, male exposure alone would not explain the sharp decrease in male survival also observed in mixed-sex vials at high mannitol concentrations. Social contact has also been posited to decrease longevity in adult flies [21], but neither males nor females showed significant differences in longevity based on culturing condition at lower mannitol concentrations (0.25 M to 0.75 M). Increased self-dosing of mannitol via increased food ingestion in order to meet the energetic demands of mating and reproduction may cause the mixed-sex vs. single-sex effect seen for both males and females in this experiment at high concentrations.

Adult sex ratios in vials of larvae raised on mannitol-containing media did not differ significantly from sex ratios produced in control vials, or from a 50–50 sex ratio. Both male and female larvae fed mannitol also had similar increases in their mean times to eclosion over increasing mannitol treatments. The delayed eclosion of pupae of both sexes and lack of a sex-biased mortality in eclosed adults may indicate significant differences in how mannitol impacts developmental stages of the same species. In addition, ~70% fewer adults of either sex were produced in the 0.8 M as compared to the 0 M mannitol treatment vials. However, because we did not control for the number of eggs laid in each vial, we cannot definitively state that mannitol reduced survival in early developmental stages.

Our data showed a significant difference in response to mannitol based on sex in adults only. An alternative hypothesis to reproductive demands is that the presence of mannitol in food signals different behaviors in adult males and females, as sexual dimorphism has been found in *D*. *melanogaster* neuronal responses to particular carbohydrates [42]. Little research has focused on mannitol perception in *D*. *melanogaster*, though mannitol receptors have been found in other insect species [8]. Gr5a sugar neurons show a small response to the administration of 100 mM mannitol [43]. Some receptors from the Gr64a-f group are also known to modulate neural responses to sugar alcohols and stimulate feeding on fermenting yeast products that may produce mannitol [3,44–46]. These neurons should be further investigated for their ability to modulate neuronal responses to mannitol and dimorphism between the sexes.

Our hypothesis that reproductive energetic demands drive mannitol ingestion and differences in mortality between sexes, and between mating vs. virgin flies, does not explain the mechanism by which mannitol ingestion kills *D*. *melanogaster* adults. This mechanism may be related to the white matter accumulated in mannitol-treated fly crops near the time of death. The lack of blue dye in the crop suggests that some food components, including the blue dye, were excreted by the flies while others remained and accumulated in the crop. If mannitol cannot be metabolized efficiently it may accumulate in the crop. *Tribolium castaneum* beetles likely utilizes NADP^+^-dependent D-arabitol dehydrogenase for mannitol catalysis [9] but this enzyme is not found in *D*. *melanogaster* [47].

Sorbitol dehydrogenase (SDH) is another possible enzymatic pathway for mannitol breakdown [48], as mannitol is an isomer of sorbitol [49]. SDH is found in *Drosophila* (Gene ID: 40836, 41313) [50]. Overloading of this pathway in other organisms leads to sorbitol accumulation in the gut [51] and the rate of mannitol catalysis by SDH is relatively low [48,52]. Expression of sorbitol dehydrogenase 1 (sodh-1) is sex-biased in *D*. *melanogaster*, with males expressing 2.86 times more sodh-1 than females [53]; increased sodh-1 expression in males may play a role in increased male survival and reduced matter accumulation on mannitol media. The presence of digestive enzymes suggest *D*. *melanogaster* and its common microbes may be able to metabolize small amounts of mannitol, but likely not the amounts consumed over the course of our experiments leading to sex-biased mortality [54–57].

Mannitol may also slow down digestion [6], elevate carbohydrate ingestion to a detrimental degree [58], or have a diuretic effect due to its slow absorption in the gut [59–60]. In flies, it is possible mannitol is transported into the hemolymph by aquaporins from the midgut [61], where it may lethally increase osmotic pressure like other sugar alcohols [62–63]. Future work should attempt to pinpoint the mechanism of mannitol’s lethality in *D*. *melanogaster*, particularly in comparison to other insect taxa where it is nutritive [9].

## Conclusions

Mannitol caused concentration-dependent decreases in longevity to 21 days in both male and female fruit flies at concentrations of 0.75 M (males) or 0.5 M (females) and above. Female longevity was more significantly decreased compared to that of males at concentrations of 0.75M and above. Actively mating males and females had decreased longevity compared to virgin males and females at concentrations of 1 M and above. Mannitol fed to larvae did not alter adult sex ratios, suggesting that sex-biased mortality due to mannitol occurs only in adults. Overall, our results support our hypothesis that sex differences in energy needs, related to the nutritional and behavioral demands of mating and reproduction, contribute to decreased longevity in females compared to males. We further conclude that both males and females have mannitol-induced decreases in longevity when mated, as compared to virgins, due to the increased costs of reproduction for both sexes.

## Supporting information

S1 Fig LC_50_ curve for adult female flies. Percent mortality of adult female flies plotted against concentration of mannitol in media. The three-parameter best-fit sigmoidal function is shown and was used to calculate the LC_50_ for flies at 21 days (0.76 M mannitol). Error bars represent one standard deviation.
(TIF)

S2 Fig Female flies fed mannitol eat more per hour than male flies fed mannitol. CAFE assay shows female flies fed 5% mannitol eat more per hour than male flies (unpaired t-test; n = 30 flies in 6 vials/sex; p = 0.0035). No difference was found in female or male consumption between sucrose-fed and mannitol-fed flies (Sidak’s MCT; female: p = 0.15, male: p = 0.80).
(TIF)

S3 Fig Adult flies housed in mixed-sex vials have decreased survival compared to flies housed in single-sex vials. Survival plot showing the significant difference in the percent survival of flies in single sex vials over mixed sex vials when given foods with the same concentration of D-mannitol. Observations were terminated at 21 days of age (n = 30 flies/sex for single sex treatments; n = 15 flies/sex for mixed-sex treatments). Males and females housed together in 1-2M mannitol treatments had much lower survival to 21 days than males or females housed in single-sex vials.
(TIF)

S4 Fig Eclosion delay in male and female fruit flies fed mannitol as larvae. Average eclosion day of male and female flies across increasing concentrations of mannitol (0 M to 0.8 M). Letters indicate highly significant differences (Mantel-Cox, p<0.001) between flies of the same sex. Error bars represent one standard deviation (n = 1,249 females; 1,181 males).
(TIF)

S5 Fig Increasing concentrations of mannitol delays eclosion. Linear regressions for male (grey circle) and female (black square) larvae showing the effect of increasing mannitol concentration on eclosion day. Both females and males eclose later when fed increasing concentrations of mannitol. Females: y = 4.034x+10.80 (F = 1620, R^2^ = 0.5650, p<0.0001), males: y = 4.271x+11.06 (F = 1168, R^2^ = 0.4977, p<0.0001). The slopes of the lines are not significantly different (F = 2.206, p = 0.1376) but the intercepts are (F = 68.38, p<0.0001). Error bars represent one standard deviation (n = 1,249 females, 1,181 males).
(TIF)
