# Supplementary figures and images for: Mannitol ingestion causes concentration-dependent, sex-biased mortality in adults of the fruit fly (Drosophila melanogaster)
Source: PLoS One. 2019 May 31;14(5):e0213760. doi: 10.1371/journal.pone.0213760 (PMC6544200; doi:10.1371/journal.pone.0213760)

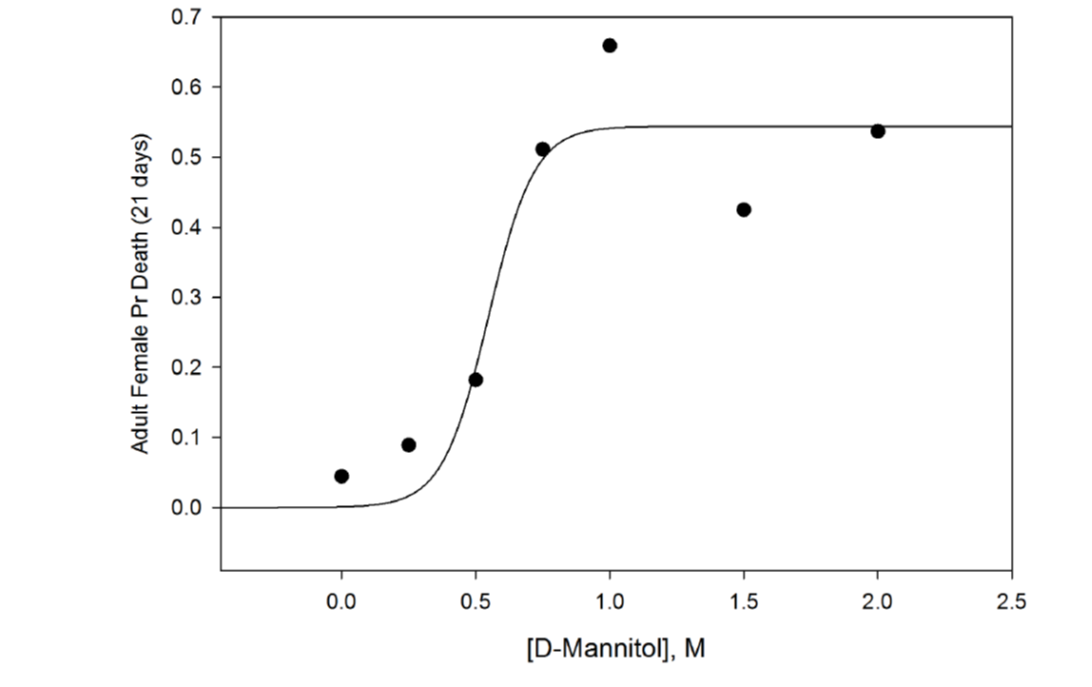

Supplement: S1 Fig — Percent mortality of adult female flies plotted against concentration of mannitol in media. The three-parameter best-fit sigmoidal function is shown and was used to calculate the LC50 for flies at 21 days (0.76 M mannitol). Error bars represent one standard deviation. (TIF) [file pone.0213760.s001.tif]

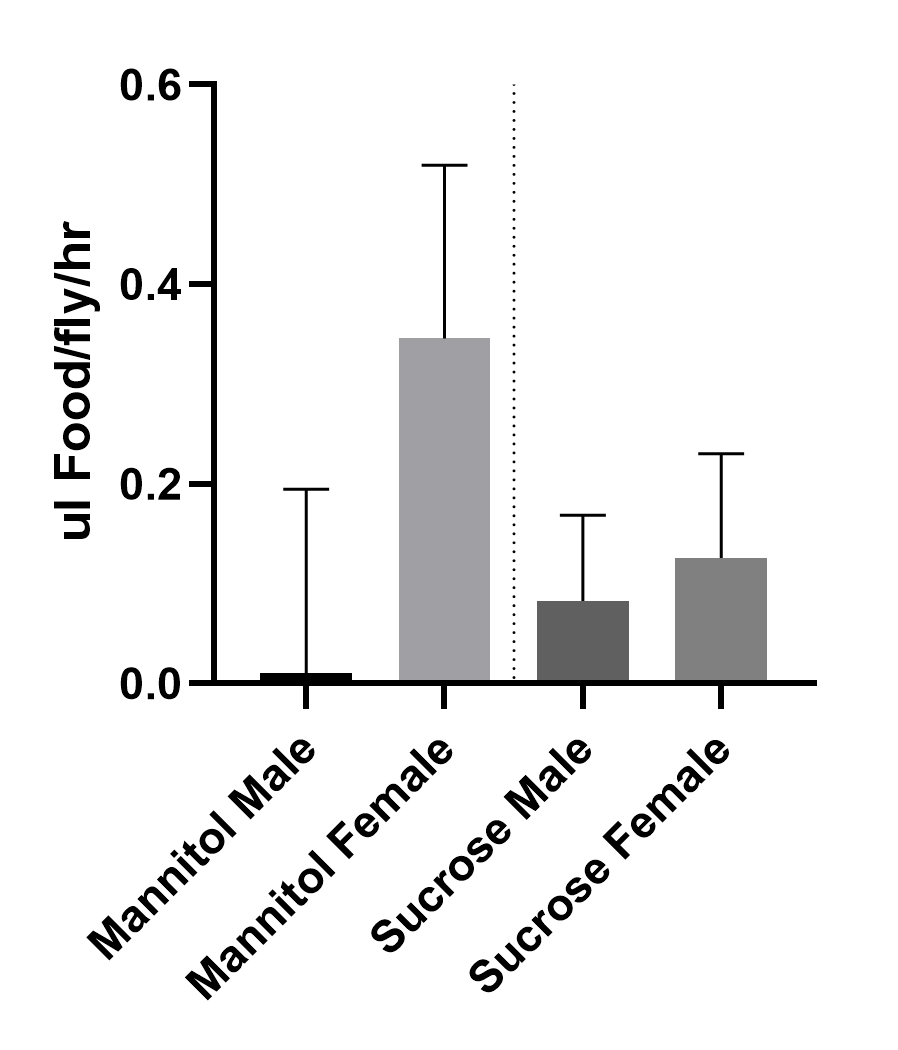

Supplement: S2 Fig — CAFE assay shows female flies fed 5% mannitol eat more per hour than male flies (unpaired t-test; n = 30 flies in 6 vials/sex; p = 0.0035). No difference was found in female or male consumption between sucrose-fed and mannitol-fed flies (Sidak’s MCT; female: p = 0.15, male: p = 0.80). (TIF) [file pone.0213760.s002.tif]

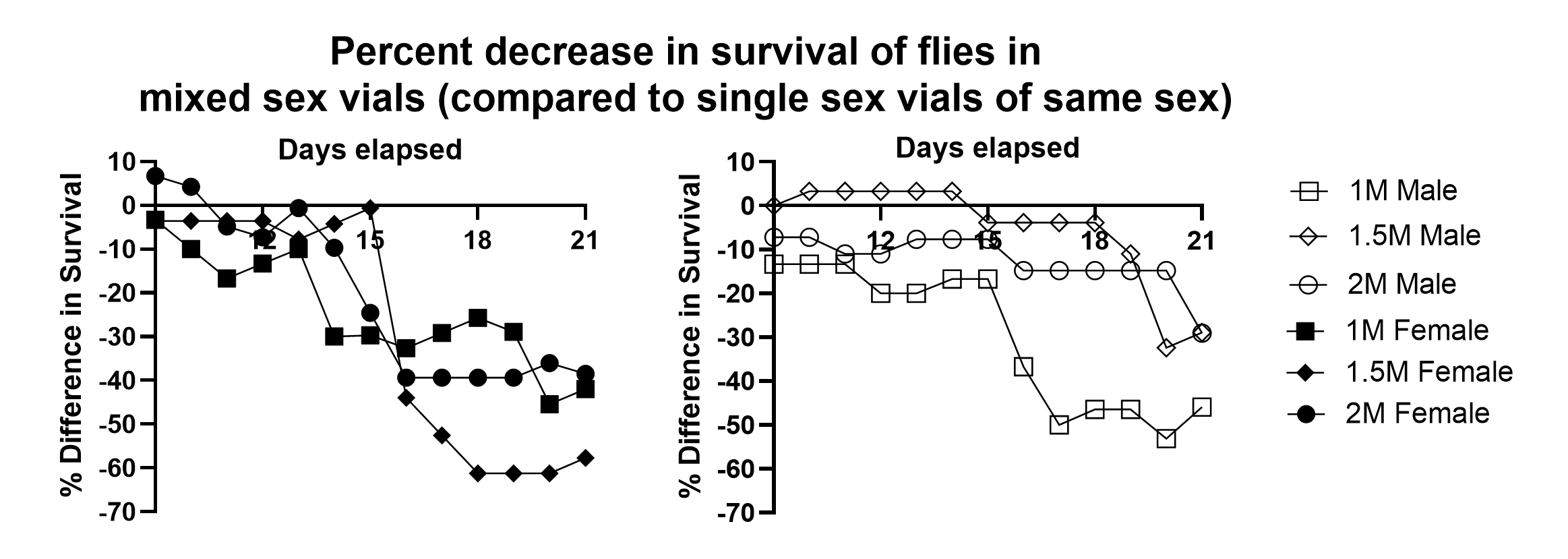

Supplement: S3 Fig — Survival plot showing the significant difference in the percent survival of flies in single sex vials over mixed sex vials when given foods with the same concentration of D-mannitol. Observations were terminated at 21 days of age (n = 30 flies/sex for single sex treatments; n = 15 flies/sex for mixed-sex treatments). Males and females housed together in 1-2M mannitol treatments had much lower survival to 21 days than males or females housed in single-sex vials. (TIF) [file pone.0213760.s003.tif]

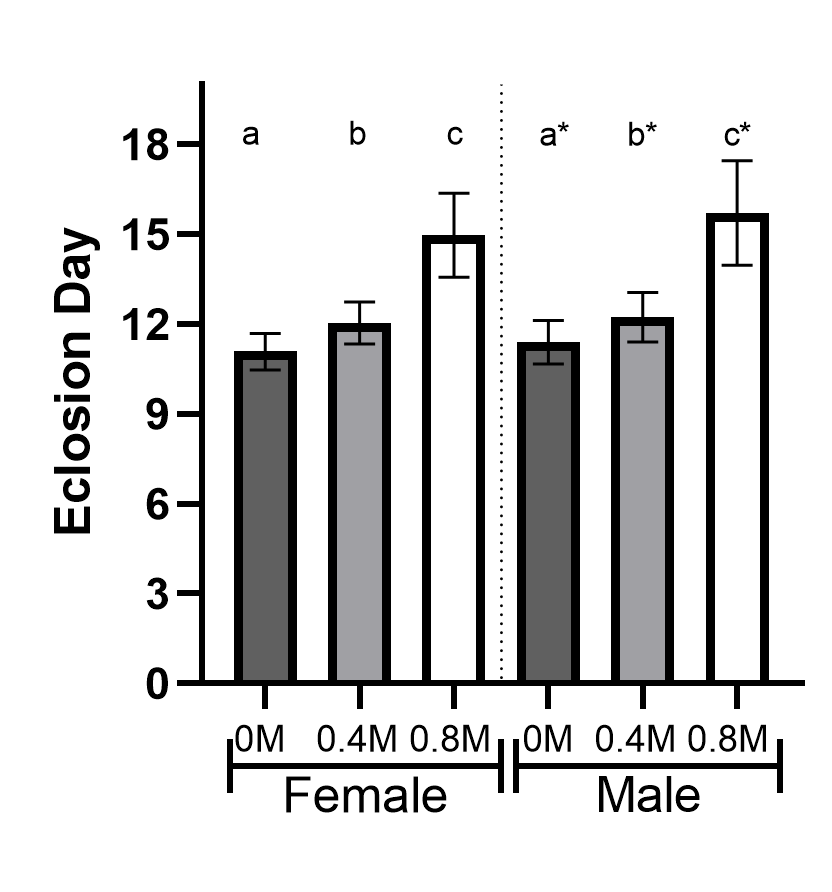

Supplement: S4 Fig — Average eclosion day of male and female flies across increasing concentrations of mannitol (0 M to 0.8 M). Letters indicate highly significant differences (Mantel-Cox, p<0.001) between flies of the same sex. Error bars represent one standard deviation (n = 1,249 females; 1,181 males). (TIF) [file pone.0213760.s004.tif]

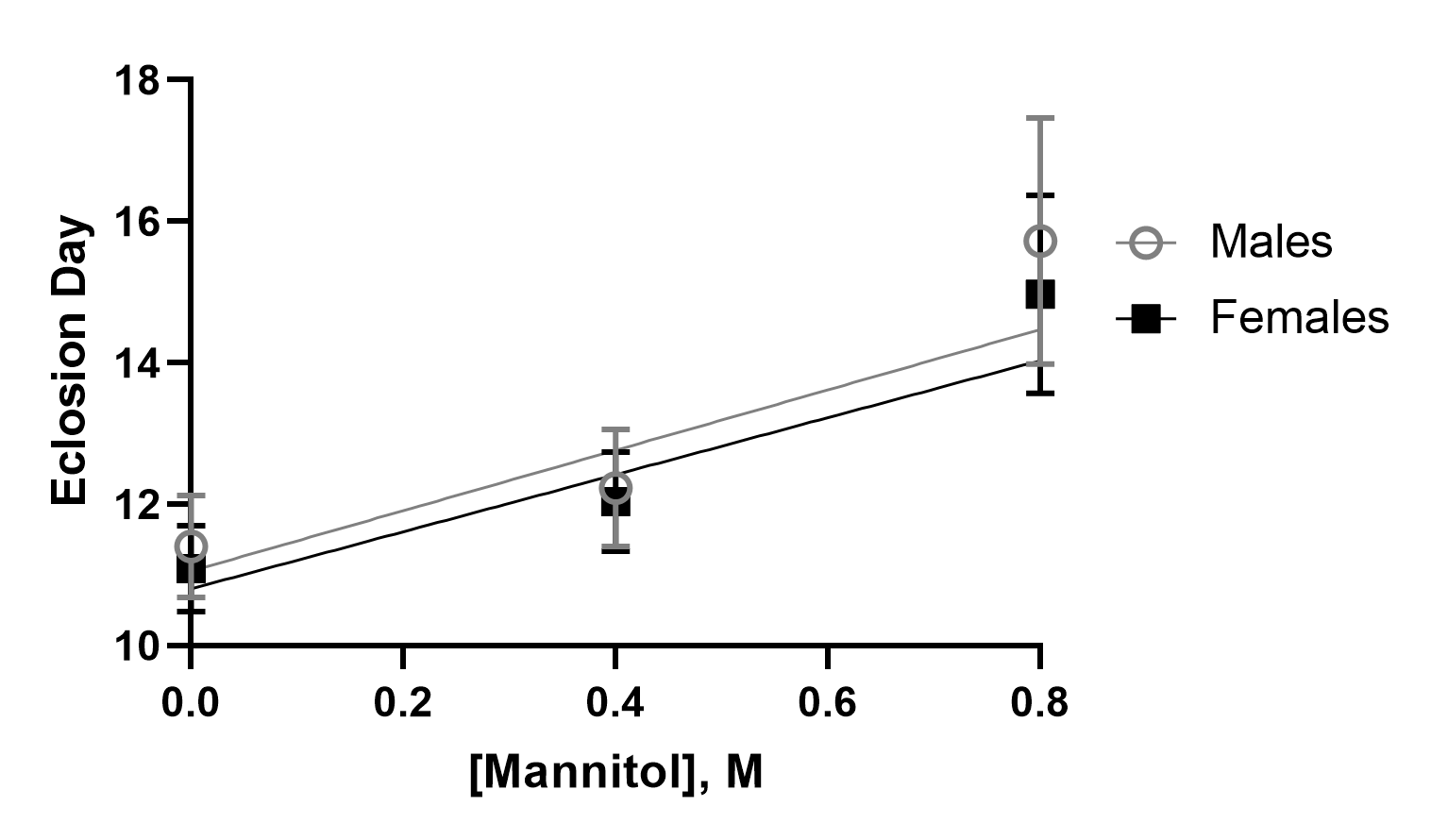

Supplement: S5 Fig — Linear regressions for male (grey circle) and female (black square) larvae showing the effect of increasing mannitol concentration on eclosion day. Both females and males eclose later when fed increasing concentrations of mannitol. Females: y = 4.034x+10.80 (F = 1620, R2 = 0.5650, p<0.0001), males: y = 4.271x+11.06 (F = 1168, R2 = 0.4977, p<0.0001). The slopes of the lines are not significantly different (F = 2.206, p = 0.1376) but the intercepts are (F = 68.38, p<0.0001). Error bars represent one standard deviation (n = 1,249 females, 1,181 males). (TIF) [file pone.0213760.s005.tif]
